# Reactive Oxygen Species Accumulation Strongly Allied with Genetic Male Sterility Convertible to Cytoplasmic Male Sterility in Kenaf

**DOI:** 10.3390/ijms22031107

**Published:** 2021-01-23

**Authors:** Yiding Liu, Bujin Zhou, Aziz Khan, Jie Zheng, Farman Ullah Dawar, Kashif Akhtar, Ruiyang Zhou

**Affiliations:** 1 College of Agriculture, Guangxi University, Nanning 530006, China; liuyiding1988@163.com (Y.L.); azizkhanturlandi@gmail.com (A.K.); summerjackk@gmail.com (J.Z.); 2 Maize Research Institute of Guangxi Academy of Agricultural Sciences, Nanning 530007, China; zhou_bujin@163.com; 3 State Key Laboratory of Cotton Biology, Institute of Cotton Research of Chinese Academy of Agricultural Sciences, Anyang 450000, China; 4 Department of Zoology, Kohat University of Science and Technology, Kohat 26000, Pakistan; farmandawar@kust.edu.pk; 5 Institute of Nuclear Agriculture Science, College of Agriculture and Biotechnology, Zhejiang University, Hangzhou 310058, China; kashif@zju.edu.cn

**Keywords:** kenaf, GMS–CMS, male sterility, reactive oxygen species (ROS)

## Abstract

Male sterility (MS) plays a key role in the hybrid breed production of plants. Researchers have focused on the association between genetic male sterility (GMS) and cytoplasmic male sterility (CMS) in kenaf. In this study, P9BS (a natural GMS mutant of the kenaf line P9B) and male plants of P9B were used as parents in multiple backcross generations to produce P9SA, a CMS line with stable sterility, to explore the molecular mechanisms of the association between GMS and CMS. The anthers of the maintainer (P9B), GMS (P9BS), and CMS (P9SA) lines were compared through phenotypic, cell morphological, physiological, biochemical observations, and transcriptome analysis. Premature degradation of the tapetum was observed at the mononuclear stage in P9BS and P9SA, which also had lower activity of reactive oxygen species (ROS) scavenging enzymes compared with P9B. Many coexpressed differentially expressed genes were related to ROS balance, including ATP synthase, electron chain transfer, and ROS scavenging processes were upregulated in P9B. CMS plants had a higher ROS accumulation than GMS plants. The MDA content in P9SA was 3.2 times that of P9BS, and therefore, a higher degree of abortion occurred in P9SA, which may indicate that the conversion between CMS and GMS is related to intracellular ROS accumulation. Our study adds new insights into the natural transformation of GMS and CMS in plants in general and kenaf in particular.

## 1. Introduction

Kenaf (*Hibiscus cannabinus* L.) is an important industrial crop that is extensively used in the paper and textile industries [1]. Its long and flexible fibers are used in clothing, car components, and padding and building materials. These fibers are environmentally friendly with potential uses in composite materials that are increasingly applied in prosthetics [2,3,4]. Recently, kenaf has been used as a phytoremediator against heavy metals [5,6]. The economic benefits, yield, and resistance of kenaf can be improved through heterosis by utilizing plants with male sterility (MS) [7].

Male sterility (MS) is the inability to produce effective pollen during sexual reproduction. MS, which has been identified in more than 320 species of plants to date [8], is a ubiquitous phenomenon that plays an important role in crop breeding and production by improving yields. Genetically, MS is divided into cytoplasmic male sterility (CMS) and genetic male sterility (GMS). CMS, which is the result of matrilineal inheritance caused by the interaction between the nucleus and mitochondria, is widely used in hybrid breeding [9,10]. Despite its advantages, there is an urgent need to resolve its shortcomings, such as limited cytoplasmic resources, low combining ability, and unstable sterility. GMS plants have many advantages, including complete sterility, safe hybrid seed production, and free combination [11], but they cannot be directly used for breeding programs because batch breeding seeds are not obtained from sterile lines. GMS can be derived from natural mutation through the manipulation of temperature and photoperiod [12,13]; however, it is difficult to obtain GMS lines through the control of a single gene [14]. Since the successful application of third-generation hybrid rice technology in 2016, studies have focused on combining GMS and CMS [15,16]. By constructing a cassette of a recombinant vector containing the two pollen-killer genes *CYP*703A3 (GMS gene) and *orf*H79 (CMS gene), enhanced rice with higher yields was obtained [17].

Occasional changes in fertility occur in the plant kingdom. In this study, we obtained the natural GMS mutant P9BS from fertile plants of P9B; after that, a CMS line with stable sterility was obtained from female P9BS and male P9B parents through multiple backcross generations. This represents the first reported natural conversion from GMS to CMS in kenaf. Some studies have reported the conversion between GMS and CMS in other species, such as nanguangzhan rice, of which a natural GMS mutant without pollen was transformed into a CMS variant [18]. After the in vitro culture of photosensitive genetically sterile rice, some plants in the F_2_ generation had CMS characteristics [19]. However, these studies only reported the phenomenon of this transformation, without further research on the molecular mechanism. This phenomenon has not been reported in kenaf, nor has the molecular mechanism been studied. Understanding how this conversion mechanism occurs is of great significance.

The tapetum has important physiological effects on pollen development. It supplies the nutrients and energy needed for the development of microspores and sporopollenin for pollen exine formation. Many studies have shown that the programmed cell death (PCD) of tapetum cells is closely related to pollen abortion [20,21,22]. In photosensitive genetically sterile rice, pollen abortion was associated with premature PCD in tapetal cells [23]. As a member of the MYB transcription factor family, AtMYB103 is necessary for tapetum development and microspore formation [24,25]. In MS lines, premature PCD in tapetum cells directly leads to pollen abortion. Additionally, reactive oxygen species (ROS) regulate many cellular functions, including PCD in plants [26]. In *Arabidopsis* and rice, excess ROS trigger the inappropriate timing of tapetal PCD [27]. However, little is known about the exact regulatory mechanism of ROS signals in the tapetum.

In previous studies, MS has been primarily attributed to pollen wall defects, microspore atrophy, anther abnormalities, and other dysfunctions related to a series of genes [12]. Some researchers have explored the mechanism of MS in kenaf. In the cytoplasmic–nuclear male-sterile line LC301A, abortion was observed to occur in the mononuclear stage [28]. However, in our field, abortion was more common in the dinuclear stage. By studying the mitochondrial gene differences of kenaf, a molecular label of the ATP8 gene was developed to distinguish fertile cytoplasm from sterile cytoplasm [29]. Several candidate genes that may cause male sterility were identified [30]. Since most of the research is a single aspect, the mechanism of kenaf MS is still unknown. In our previous study, GMS in HMS/722HA plants was converted to CMS through the creation of a transgenic mutant; however, its molecular mechanism was not elucidated [31]. Accordingly, in this study, we obtained the GMS material P9BS as a natural mutant, and after seven backcross generations with P9B, the stable CMS line P9SA was established. Then, we explored the differences in the characteristics of the three kenaf lines, including pollen abortion and candidate fertility-related genes. The cytological morphologies of the anthers and the transcriptomes of the three kenaf lines (P9B, P9BS, and P9SA) via RNA-Seq were evaluated. This study also included a comparative analysis between CMS and GMS by evaluating physiologies and molecular interactions to better understand GMS and CMS transformations. The knowledge gained from this study provides a foundation for potentially creating new hybrid breeding systems of kenaf.

## 2. Results

### 2.1. Phenotypic and Cytological Characterization of P9B, P9BS, and P9SA

**Figure 1:**
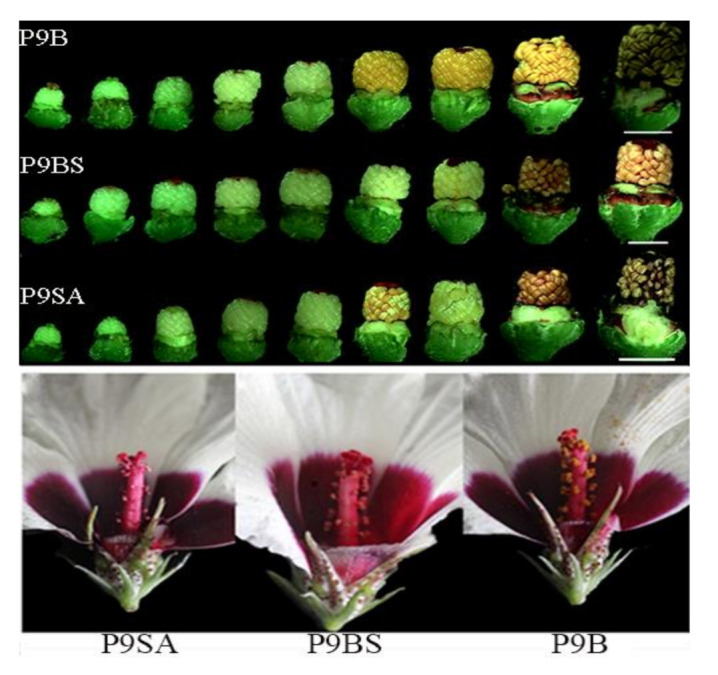
Comparison of the morphological characteristics among P9SA, P9BS, and P9B. Bar = 0.5 cm.

There were no significant differences among the three flower structures, except for the anther color (Figure 1). However, there were noticeable differences in the anther morphologies of P9B, P9BS, and P9SA. Normal anthers were observed in P9B, which were plump, and pollen grains were released at dehiscence. P9BS anthers were plump but not at all dehiscent, while P9SA anthers were shriveled (Figure 1).

**Figure 2:**
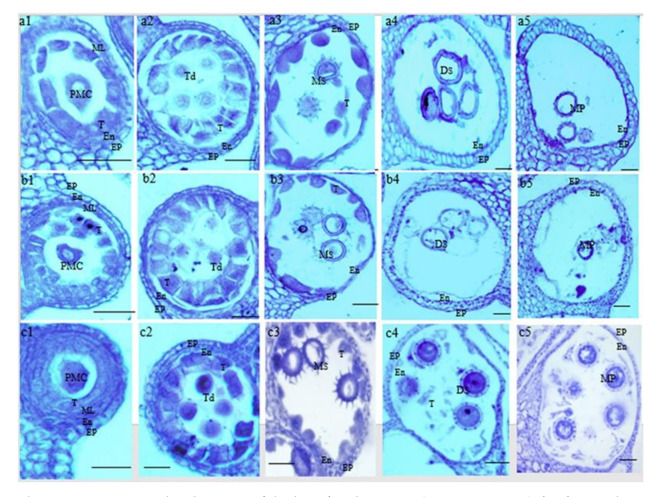
Microspore development of the kenaf anthers in P9SA (**a1**–**a5**), P9BS (**b1**–**b5**), and P9B (**c1**–**c5**). Bar = 20 μm. Ep, epidermis; En, endothecium; ML, middle layer; T, tapetum; Ms, microspore; MP, mature pollen; PMC, pollen mother cells; Td, tetrad microspore; Ms, mononuclear microspore; Ds, dinuclear microspore; MP, mature pollen grain.

In order to determine the period and mode of abortion during different anther development stages, paraffin sections were produced to compare the cytological features of P9B, P9BS, and P9SA. No noticeable differences between P9B, P9BS, and P9SA were observed at the pollen mother cell stage. During the meiotic stage of pollen mother cells, the anther wall was divided into the epidermis, endodermis, middle layer, and tapetum (Figure 2(a1,b1,c1)). At the tetrad stage, tapetum cells expanded laterally and radially and increased in size in P9B, P9BS, and P9SA; however, the tapetum in P9BS and P9SA was more vacuolized than that in P9B (Figure 2(a2,b2,c2)). At the mononuclear stage, the microspore cells were round or oval in shape and squeezed near the cell wall, and the tapetum started to gradually degrade. P9BS and P9SA tapetum cells had irregular shapes, and their degradation rates were higher than that of P9B. Additionally, the cell walls of P9SA and P9BS were thinner than those of P9B (Figure 2(a3,b3,c3)). In P9B, abundant cytoplasm and nutrients for pollen development began to fill microspores at the dinuclear stage. In contrast, the contents of the P9BS and P9SA microspores were markedly reduced compared with those of P9B; only a few shrunken and misshapen pollen grains were observed in P9SA and P9BS. In addition, the P9SA pollen grains were visibly hollow, and generative cells had incomplete exine walls, whereas, in P9BS, generative cells with detached exine walls were observed (Figure 2(a4,b4,c4)). During the mature pollen grain stage, P9B formed fully developed and nutrient-rich pollen. In contrast, P9BS and P9SA did not produce normal pollen, and the anther locules were nearly empty: only hollow pollen cells and some remnants of tapetal cells were observed (Figure 2(a5,b5,c5)). The observation of paraffin sections revealed that pollen abortion in P9SA and P9BS occurred at the dinuclear stage due to defective exine wall formation, which directly affected the normal pollen development process.

### 2.2. De Novo Assembly and Sequence Annotation

**Figure 3:**
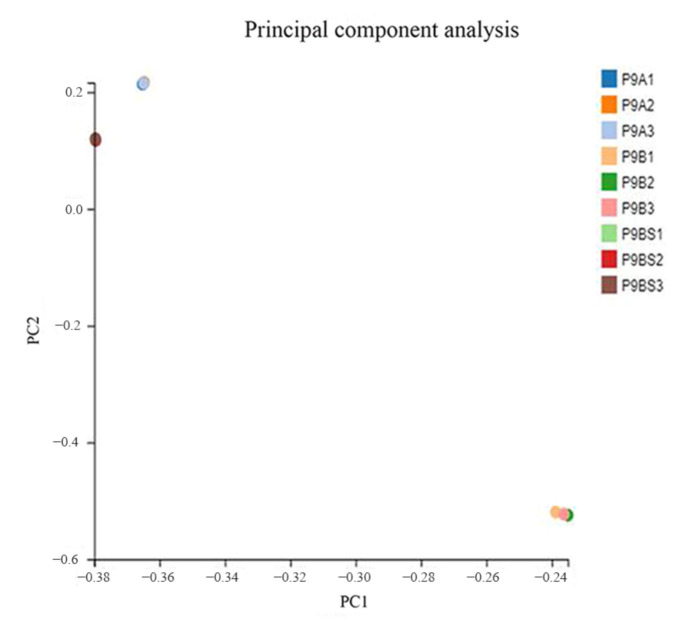
Principal component analysis of the three kenaf lines.

**Figure 4:**
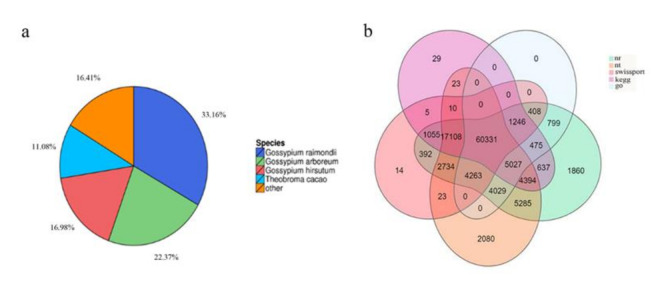
(**a**) The species distribution of the annotated unigenes. (**b**) Venn diagram of results from NR, NT, KEGG, SwissProt, and GO databases.

RNA-Seq analysis using the BGISEq-500 platform produced a total of 59.06 GB of clean data, including 19.59 GB from P9SA, 19.86 GB from P9BS, and 19.61 GB from P9B, from 9 libraries. To ensure reliability, principal component analysis (PCA) was performed, and each sample was tightly clustered, indicating the high repeatability of the sequence data. The three lines were divided into two separate groups, suggesting a close similarity between P9SA and P9BS (Figure 3). All of the reads obtained from the three kenaf lines were de novo assembled. A total of 588,381 transcripts (N50:1862), with an average length of 1281 bp, and 125,778 unigenes (N50:2325), with an average length of 1728 bp, were identified (Table 1). A total of 98,344 CDSs were detected by Transdecoder (version v3.0.1, https://transdecoder.github.io). The length distribution of all assembled kenaf genes showed that 16.62% of CDSs were longer than 2000 bp, and the majority of the CDSs were shorter than 1500 bp. Among the unigenes, 34.25% were longer than 2000 bp, and the number of unigenes was evenly distributed in each length. After comparing unigenes with seven functional databases, 110,043 (NR: 87.49%), 105,307 (NT: 83.72%), 87,589 (SwissProt: 69.64%), 91,815 (KOG: 73.00%), 90,340 (KEGG: 71.82%), 76,578 (GO: 60.88%), and 92,286 (Pfam: 73.37%) unigenes were functionally annotated (Figure 4b). Sequence alignment was performed to determine the distribution of sequence matches to other species; of the aligned sequences, 33.16% matched those from *Gossypium raimondii*, 22.37% matched *Gossypium arboretum*, 16.98% matched *Gossypium hirsutum*, 11.08% matched *Theobroma cacao*, and 16.41% matched other species. The species distribution of the annotated unigenes is shown in Figure 4a.

### 2.3. Identification of the DEGs among Different Kenaf Lines

**Figure 5:**
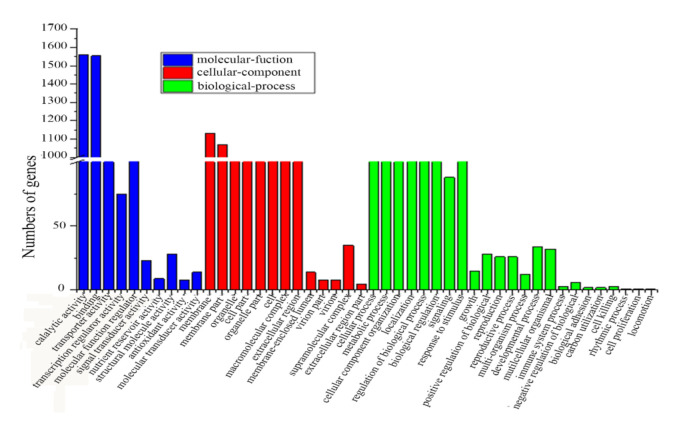
Gene ontology classification of DEGs among the three different kenaf lines.

**Figure 6:**
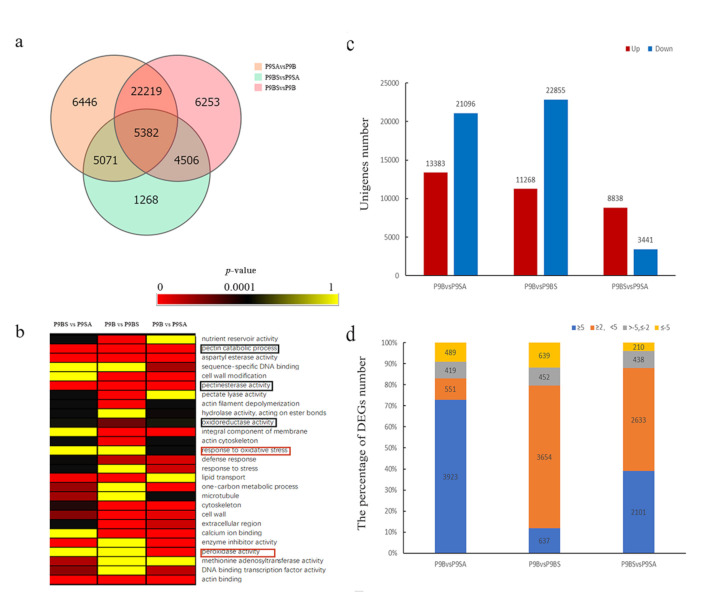
(**a**) A Venn diagram showing the numbers of DEGs resulting from three comparisons: P9BS vs. P9SA, P9B vs. P9SA, and P9B vs. P9BS**.** (**b**) GO enrichment analysis of DEGs from the three comparisons. The *p* values of GO terms in the three comparisons are shown in the heatmap. The bar indicates significance values. The black outline indicates the three terms related to pollen exine wall and reactive oxygen species (ROS) among the three lines. The red outline shows the two unique terms involved in ROS in the comparison of P9B vs. P9SA. (**c**) The numbers of up- and downregulated unigenes in the three comparisons. (**d**) The distribution of the (log_2_ fold changes in DEGs between P9B, P9SA, and P9BS.

Differentially expressed genes (DEGs) were identified by performing pairwise comparisons. Further analysis identified a total of 5382 co-expressed DEGs (fold change ≥ 2.00 and adjusted *p* value ≤ 0.05), among which 3135 genes had annotation information in the GO database. The GO classification analysis showed that genes involved in catalytic activity (1561), binding (1554), and the membrane (1133) were relatively abundant (Figure 5). The numbers of DEGs in each pairwise comparison are shown in a Venn diagram (Figure 6a). In the comparison of P9B vs. P9SA, 13,383 genes were upregulated and 21,096 genes were downregulated. In the P9B vs. P9BS comparison, 11,268 genes were upregulated and 22,855 genes were downregulated, whereas 8838 upregulated genes and 3441 downregulated genes were identified in the comparison of P9BS vs. P9SA (Figure 6c). GO functional enrichment was performed for further analysis of DEGs, and 62 terms were significantly enriched in the comparison of P9BS vs. P9SA (*p* < 0.05). In the comparison of P9B vs. P9SA, 42 terms were significantly enriched, while 34 terms were significantly enriched in the comparison of P9B vs. P9BS (Tables S2–S4). Twenty-seven coexpressed or unique terms were chosen for analysis to understand the relationship between male sterility and DEGs (Figure 6b). In the comparisons of P9B vs. P9SA and P9BS vs. P9SA, most DEGs had increased expression levels, with log_2_ fold changes greater than 5, while, in the comparison of P9BS vs. P9SA, they ranged from 2 to 5. The results of these pairwise comparisons indicate that the expression levels of multiple genes considerably differ among the three plant lines (Figure 6d).

### 2.4. DEGs Related to ROS

**Figure 7:**
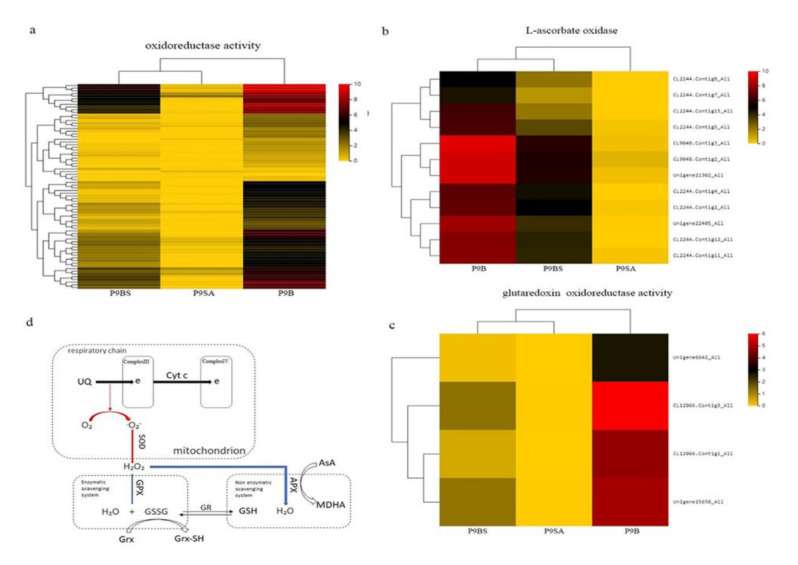
(**a**) Heatmap of coexpressed DEGs related to oxidoreductase activity in P9B, P9BS, and P9SA. (**b**) Heatmap of genes related to glutaredoxin. (**c**) Heatmap of genes encoding L-ascorbate oxidase. The color scale indicates the log_2_ (FPKM + 1) values. (**d**) Overview of the ROS production and scavenging pathway.

In the present study, coexpressed DEGs were significantly enriched in oxidoreductase activity terms (GO:0016491, Figure 6b). Among the DEGs, a series of genes and KEGG pathways were involved in ROS clearance (Figure 7a), including genes related to peroxidase (POD) and superoxide (SOD). We identified three genes related to POD, which were very highly expressed in P9B, highly expressed in P9BS, and downregulated in P9SA (Unigene15808_All, Unigene6255_All, and CL4172.Contig15_All). SOD4 (CL5810.Contig4_All), an important gene in the first step of ROS scavenging [32], was expressed at the same levels as those of the identified peroxidase genes. Genes associated with the mitochondrial respiratory chain, the tricarboxylic acid cycle (TCA), and ATP synthase, which play important roles in ROS production, were also significantly different [33,34]. The TCA cycle plays a vital role in many metabolic pathways, including energy metabolism, while ROS can damage TCA-related enzymes, leading to metabolic abnormalities [35]. Ten genes associated with the TCA cycle were upregulated in the comparisons of P9B vs. P9SA, P9B vs. P9BS, and P9BS vs. P9SA. The expression of five ATP synthase-related genes (which encode mitochondrial proton-transporting ATP synthase complex and coupling factor F0) with important functions in ROS generation [36,37] decreased in the order of P9B, P9BS, and P9SA. Ubiquinol-cytochrome-c reductase (complex III, Unigene12642_All) and two cytochrome-c oxidase genes (complex IV, CL7398.Contig6_All, CL7398.Contig5_All), which cause electrons to bind directly to oxygen molecules and produce an excess of ROS [38,39], had the highest expression in P9B, the second highest expression in P9BS, and the lowest expression in P9SA in our study (Table S5).

A non-enzymatic protection mechanism directly involved in ROS clearance plays an essential role in maintaining cell redox homeostasis [40,41]. Small-molecular-weight antioxidants, such as ascorbic acid and glutathione, are directly involved in ROS elimination in non-enzymatic protection mechanisms. Additionally, glutaredoxin (Grx) catalyzes the reduction of peroxides, such as hydrogen peroxide, and is the main enzyme that prevents plant cells from being damaged by oxygen [42,43,44]. In this study, several genes that participate in ascorbate oxidase and Grx metabolism were identified through KEGG pathway analysis. Ascorbic acid (AsA) not only acts as a reducing agent to remove hydrogen peroxide but also provides a reducing force for the ROS enzymatic scavenging system. The expression levels of 12 genes that encode L-ascorbate oxidase were higher in P9BS and P9B, and they were markedly different from those in P9SA. Further comparison between P9BS and P9B showed that although the expression of these genes in P9B was higher than that in P9BS, there was no significant difference between the two (Figure 7b). Four Grx genes were found among the coexpressed DEGs. In contrast to the expression patterns of L-ascorbate oxidase genes, the expression of Grx genes in P9B was significantly different from that in P9SA and P9BS. By comparing P9BS and P9SA, we found that the expression levels of these genes were very low in P9SA (log_2_(FPKM + 1) < 1, Figure 7c).

### 2.5. DEGs Related to the Pollen Development Process

**Figure 8:**
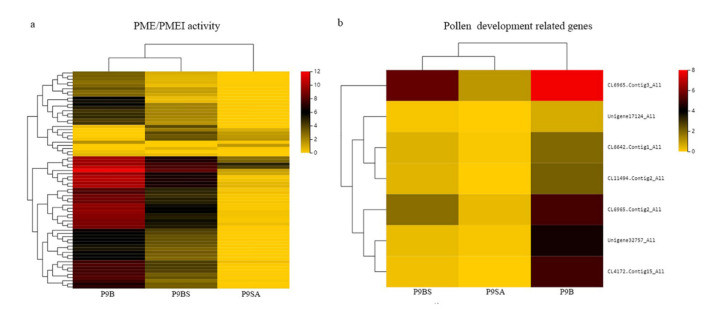
(**a**) Heatmap of coexpressed DEGs related to PME/PMEI in three lines of kenaf. (**b**) Heatmap of coexpressed DEGs related to pollen development and expressed specifically in pollen. The color scale indicates the log_2_ (FPKM + 1) values.

Based on the transcriptome analysis of coexpressed DEGs, two pectin metabolism-related GO terms—pectin catabolic process (GO:0045490) and pectinesterase activity (GO:0030599)—were identified. As is well known, pectinesterase (PME) and pectin methylesterase inhibitor (PMEI) play important roles in plant pollen wall formation and pollen development [45,46,47]. The specific pattern of pollen walls in flowering plants is crucial to the appropriate development of pollen and its normal fertility. From the analysis, 69 genes related to PME/PMEI (Figure 8a) and 90 pectin metabolism-related genes were identified, which were significantly enriched in two enzymes (K01051//pectinesterase [EC:3.1.1.11], K01728//pectate lyase [EC:4.2.2.2]) and involved in one pathway (Ko00040//Pentose and glucuronate interconversions). PME/PMEI-related gene expression levels in the three kenaf materials were compared and analyzed, and the results showed that P9SA was significantly different from P9BS and P9B. Further, their expression in P9B was slightly higher than that in P9BS. Seven genes involved in pollen development and expressed specifically in pollen were identified. The specific expression pattern is shown in Figure 8b. Among these genes, two (CL6965.Contig2_All and CL6965.Contig2_All) were related to pollen exine formation (GO:0010584), which encode TPR (tetratricopeptide repeat) proteins. TPR genes are widely involved in biological processes such as cell cycle regulation, development modulation, and pollen germination [48,49]. Their expression levels in P9B were higher than those in P9BS, while P9A had the lowest expression. Three genes (CL6642.Contig1_All, Unigene17124_All, and Unigene32757_All) encoding K04733//interleukin-1 receptor-associated kinase [EC:2.7.11.1], including RLK (Receptor-Like kinase) and Rks (Receptor kinase), were identified. Previous studies have shown that Rks and RLK play crucial roles in transmembrane signaling and the control of plant reproduction, growth, and development, which includes the regulation of the MAPK cascade, NADPH oxidase, and ROS [50,51,52,53]. Their expression pattern was different from that of TPR genes; P9B had a higher expression, while there was little difference between P9SA and P9BS.

### 2.6. Transcription Factor Related to Male Sterility

**Figure 9:**
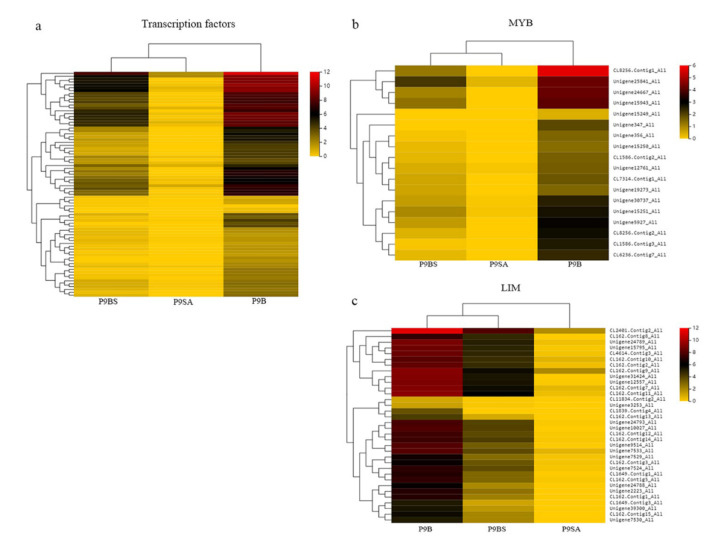
(**a**) Expression profiles of transcription factor-encoding genes related to male sterility are shown in a heatmap. (**b**) MYB expression levels among coexpressed DEGs. (**c**) Heatmap of coexpressed DEGs related to the LIM transcription factor family. The color scale indicates the log_2_ (FPKM + 1) values.

A total of 97 coexpressed DEGs with the ability to encode TFs were divided into 18 families. Eighteen genes were downregulated and 79 genes were upregulated (Figure 9a), of which 5 categories were all upregulated (MYB, MADS, LIM, C2C2-GATA, and bHLH). The five TF family genes regulate a series of biochemical processes, including floral organ development, the abnormal degradation of the tapetum, and cellular antioxidants [54,55,56]. There were 18 MYB family genes, 34 LIM family genes, 5 MADS-box genes, 1 C2C2-GATA family gene, and 1 bHLH family gene (Figure 9b,c). The results showed that the expression of genes in the MYB family was significantly higher in P9B and very low in P9SA (the value was close to zero) compared with P9BS (the values range from 2 to 5). Unigene12761_All, Unigene15249_All, Unigene15250_All, and Unigene15251_All are peroxide-induced TF (MYB) genes that play important roles in ROS regulation. Their expression levels in P9B and P9BS were similar, but they were quite different from those in P9SA.

### 2.7. qRT-PCR Confirmation of DEG Data

**Figure 10:**
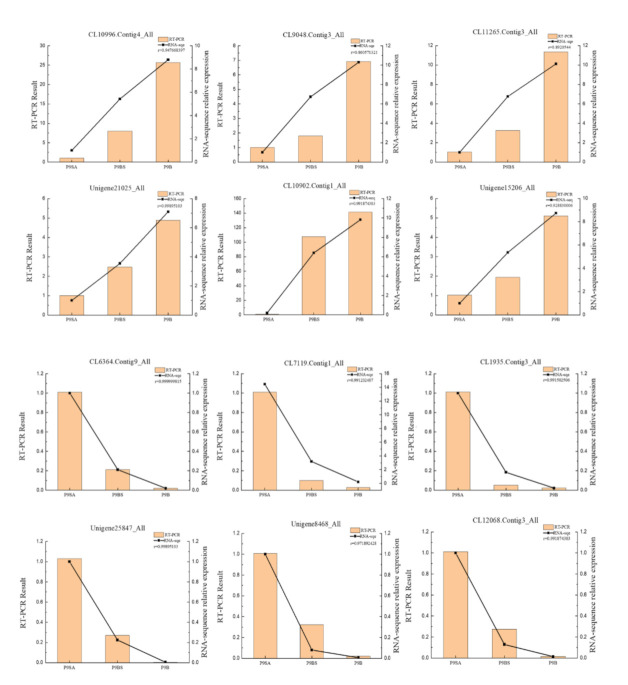
RT-qPCR verification results of 12 genes. The relative expression levels of the 12 genes were calculated using the 2^−∆∆*C*t^ method. r: Represents the correlation coefficient between RT-qPCR data and RNA-seq data based on the Correl function.

To verify the expression levels of differentially expressed genes identified in the three kenaf lines, we chose 12 genes (6 upregulated and 6 downregulated) randomly for qRT-PCR validation to confirm the accuracy of the sequencing results (Figure 10). Overall, the qRT-PCR expression trends of the 12 genes were consistent with the results of RNA-Seq.

### 2.8. The Activity of Enzymes Involved in Reactive Oxygen Scavenging Pathways

**Figure 11:**
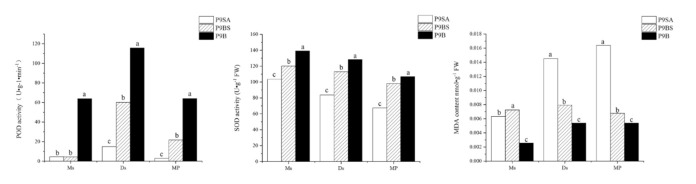
(**a**) POD activities in the anthers of P9B, P9BS, and P9SA; (**b**) SOD activities in the anthers of P9B, P9BS, and P9SA; and (**c**) MDA contents in the anthers of P9B, P9BS, and P9SA; Ms, mononuclear stage; Ds, dinuclear stage; MP, mature pollen grain stage. Significant differences were assessed by Duncan’s method using SPSS.

GO functional enrichment and KEGG pathway analysis of coexpressed DEGs identified many genes related to ROS, which suggests that the ROS metabolism pathway might be abnormal in P9SA and P9BS. In order to validate this hypothesis, we selected two enzymes (SOD and POD) involved in ROS clearance to measure their activity in the three kenaf anthers; malondialdehyde content was also measured at the same time. SOD activity was higher in P9B than in P9BS and P9SA and significantly different from that in P9SA at all three anther development stages. However, at the dinuclear stage (Ds), which is involved in pollen sterility, the difference in SOD activity was not significant between P9B and P9BS. We found that the activity of POD first increased and then reached its maximum at the Ds stage, after which it decreased in all three kenaf lines, while POD activity in P9B was markedly higher than that in P9BS and P9SA. Furthermore, at the Ds stage, the POD activity was significantly different between P9B, P9BS, and P9SA, and the value for P9SA was always very low. In addition, the MDA content in P9SA was considerably higher than that in P9B and P9BS. The MDA content had an increasing trend from the Ms to MP stage, except for in P9BS. This is an interesting result because the trend of MDA content was opposite to that of SOD activity at the key stage of pollen abortion (Figure 11).

## 3. Discussion

The natural occurrence of GMS in plants is not uncommon, but the natural transformation from GMS to CMS is a rare phenomenon. Exploring the causes and mechanism of this phenomenon is of great value for innovative breeding systems. In the present study, three lines of kenaf from the same source were analyzed: fertile P9B, the natural GMS mutant P9BS, and the converted CMS line P9SA. These materials are considerably advantageous for studying the mechanisms of sterility and the transformation from GMS to CMS.

### 3.1. Importance of Materials for RNA-Seq Analysis

Due to the lack of a kenaf reference genome, few transcriptome studies on kenaf male sterility have been conducted. Furthermore, the sources of the fertile and sterile materials involved in previous comparative analyses have had quite different genetic backgrounds [31,57]. However, in the present study, the transcriptomes of three plant materials with cytoplasmic and nuclear homology were investigated. While the analysis of the transcriptome of the CMS cotton line LD6A [58] was similar to our study in terms of materials selection, the genetic differences between the plants were still significant. The characteristics of the materials in the present study provide better conditions for identifying key genes related to male sterility in plants.

### 3.2. Production and Clearance of ROS in Anther Development

ROS are important signaling molecules involved in many biochemical processes for the normal growth and development of plants. For example, the *MAPK* signaling pathway, which controls responses to various stresses, is activated by H_2_O_2_ in *Arabidopsis* [59]. Furthermore, excess ROS can damage protein structures and act as toxins that kill cells [60]. According to previous studies, the mitochondrion is a vital organelle for respiration and is a major site of ROS production via the electron transport chain [38,61]. Several genes encoding Ubiquinol-cytochrome-c reductase (complex III) and cytochrome-c oxidase (complex IV), which play important roles in the respiratory chain, were identified (Figure 7d). The expression patterns of coexpressed DEGs associated with the TCA cycle, which provides NADH/FADH for mitochondrial respiration, were consistent. The expression levels of these genes were higher in P9B and lower in the male-sterile lines P9BS and P9SA. Mitochondrial respiratory complex chain III contributes to ATP synthesis, and its dysfunction can limit ATP production [62]. In general, the expression of ATP synthase is lower in the male-sterile lines. In Honglian rice, *orf*H79 was observed to combine with complex III to damage the structure of mitochondria and thus lead to CMS [63]. In the present study, the expression levels of ATP synthase-related genes among coexpressed DEGs in male-sterile P9BS and P9SA were markedly lower than those in P9B. The results show that in P9BS and P9SA, the electron transport chain may be suppressed due to the inhibition of enzymes in the oxidative respiratory chain; hence, excess ROS were produced and damaged ATP synthase-related mtDNA, which directly led to the inhibition of ATP production. The energy necessary for the development of microspores was thus insufficient due to the impaired mitochondria. The expression levels of related DEGs were much lower in P9SA compared with those in P9BS, and they likely significantly contributed to the higher degree of microspore abortion in P9SA (Figure 2).

Plants must counteract oxidative stress caused by excess ROS through an efficient scavenging system in order to avoid oxygen-induced damage. There were two types of ROS scavenging systems (Figure 7d): enzymatic and non-enzymatic [64]. The enzymatic system involves several antioxidative enzymes, including POD, SOD, and CAT, while the non-enzymatic system includes major antioxidants such as ascorbate (AsA) and glutathione (GSH). In our study, the expression levels of DEGs related to POD and SOD were significantly downregulated in P9BS and P9SA. In the non-enzymatic system, the expression levels of AsA- and Grx-related genes were lower in male-sterile lines. Further verification showed that at the mononuclear and dinuclear stages, MDA decreased in the order P9SA, P9BS, and P9B, whereas the activities of SOD and POD were highest in P9B among the three lines. Thus, the physiological data were consistent with the RNA-Seq analysis. In addition, the expression trends of four predicted peroxide-related MYB family transcription factors, which are responsive to oxidative stress, were similar to those of genes involved in the ROS scavenging system. The results suggest that the imbalance between ROS accumulation and clearance causes the cells to produce excess ROS, the levels of which were highest in the sterile line P9SA. This may be a significant reason for the higher degree of abortion in CMS lines transformed from GMS.

### 3.3. Burst of Reactive Oxygen Species Causes Premature PCD of Tapetum Cells and Abnormal Pollen Wall Development

The anther plays a key role in providing a suitable environment for pollen development, which directly determines the fertility of plants. During the process of pollen development, tapetum cells have various physiological functions. The tapetal cells timely enter the PCD process and release a large number of nutrients and structural substances such as sugars, lipids and proteins into the pollen sac to ensure the normal development of microspore and successful pollination [65]. The specific expression of MYB80 in tapetum and microspores leads to abnormal tapetum degradation and failure to provide nutrients to microspores, leading to male sterility [66]. The DEFECTIVE POLLEN WALL (DPW) is a nuclear gene encoding lipid acyl reductase for fat metabolism. The gene is expressed specifically in the tapetum and microspores, and its main function is to catalyze the hydroxylation of C16 and C18 fatty acids, the *dpw* mutant showed defects in the pollen outer wall, leading to microspore abortion [67]. The normal development of tapetal cells is the basis of pollen fertility, and abnormal programmed cell death (PCD) of tapetal cells, including advanced or delayed death, can lead to pollen abortion. In previous studies, because of the imbalance in the supply and consumption of ROS, excess ROS accumulated and led to an oxidative burst, followed by pollen abortion [39,68]. In CMS-WA rice, WA352 was found to interact with COX11 and thereby trigger an ROS burst that contributed to PCD, resulting in the development of CMS [69]. Due to excess ROS, PCD in the tapetum increased, which caused male sterility in rice MADS3 [70]. Similarly, in our study, the tapetum cells in male-sterile lines had developmental anomalies starting in the mononuclear stage, leading to premature tapetum degradation with a relatively high level of MDA content compared with P9B. At the same time, the CMS line P9SA had the highest content. The microspore is in an environment that is rich in ROS; thus, noticeable deformities and slight rates of degradation were observed in P9BS and P9SA, while compared with P9BS, P9SA has a higher rate. Moreover, at the dinuclear stage, the tapetum was completely degraded, resulting in a lack of nutrients for microspores. Paraffin sections revealed that the microspores that developed in P9SA were more deformed than those in P9BS. It is likely that the adverse effects of ROS on P9SA are more pronounced due to a higher accumulation of ROS and greater oxidative damage.

The integrity of the pollen wall is a necessary condition for the normal development of pollen and microspores, and it is dependent on tapetum cells releasing sporopollenin and forming the exine wall of pollen. In our study, paraffin sections revealed a lack of pollen walls in P9BS and P9SA at the Ds stage (Figure 2(a4,b4)), which was likely caused by the abnormal PCD in the tapetum. In addition to the tapetum cells, pectin metabolism also plays an important role in pollen development [71,72]. In *Arabidopsis*, 18 of 67 PME genes are highly expressed during the stages of pollen development [73]. The expression patterns of PME genes are significantly different between fertile and sterile buds; for example, they are not expressed in the male-sterile line of onion [74]. This agreed with the results of our study. Among the coexpressed DEGs, those involved in pollen wall formation, PME/PMEI, and RLKs were assayed, and their expression levels in P9BS and P9SA were lower than those in P9B. The decreased expression of these genes led to incomplete and deformed pollen exine in P9BS and P9SA, as the completeness of the pollen wall depended on their expression. Between these two lines, the pollen in P9SA had a higher degree of abortion than that in P9BS.

## 4. Materials and Methods

### 4.1. Plant Materials

In the present study, male-sterile plants (P9BS) were produced from the kenaf line P9B. These plants were shown to have fertile cytoplasm via molecular tag detection developed in our previous studies [29] (Figure S1). P9B was used as the male parent for strict backcrossing to harvest seeds. The fertility of progeny plants in the F1 generation obtained from the sown seeds conformed to the genetic law of GMS, and the ratio of fertile to sterile plants in the F2 generation was 1:1. Thereafter, male-sterile plants were used as female parents and P9B served as male parents for backcrossing (7 generations), which produced all sterile progenies, named P9SA (Table 2). All kenaf seeds were sown in soil under the same conditions at Guangxi University, Nanning, Guangxi, China. According to a previous method [28], floral buds with lengths of 1–2.5 mm, 2.5–3.5 mm, 3.5–5 mm, 5–7 mm, and 7–15 mm (corresponding to the pollen mother cell stage, tetrad stage, mononuclear stage, dinuclear stage, and mature pollen grain stage) were collected. After morphological and cytological observations, floral buds were collected at the dinuclear stage (5–7 mm) after removing the petals. Pollen in the abortion stage (dinuclear stage, 5–7 mm in diameter) was frozen in liquid nitrogen and stored at −80 °C for RNA isolation.

### 4.2. Morphological and Cytological Observations

The flowers of all lines were observed using a digital camera (Canon750D, Japan) at the full bloom stage. Flower morphology was observed at different stages with a stereomicroscope (Olympus, Japan). The flower buds were stripped of sepals and petals, placed in a 4 °C Carnot fixative solution for 24 h, and then dehydrated with gradient alcohol (75%, 80%, 85%, 90%, 95%, and 100%) for 1 h per wash. The dehydrated floral buds were embedded in paraffin wax at 58 °C. After three weeks, the tissue embedded in paraffin was cut into 10 µm pieces and placed on glass slides, stained with iron vanadium hematoxylin, and observed and photographed under an inverted microscope (Leica, Germany).

### 4.3. Total RNA Extraction, cDNA Library Construction, and Deep Sequencing

The total RNA from the anther of each sample was extracted using a Quick RNA Isolation Kit (TransGen Biotech, Beijing, China) according to the manufacturer’s protocol. The RNA concentration and quality were measured using a NanoDrop 2000 spectrophotometer (Thermo Scientific, Waltham, USA). An Agilent 2100 Bioanalyzer (Agilent RNA 6000 Nano Kit) was used to detect total RNA concentration, RIN value, 28S/18S, and fragment size. In each RNA sample, the same amount of total RNA was used for the transcriptome library workflow using the BGISEQ-500. The total RNA was treated by enriching mRNA polyA tails with OligodT magnetic beads. Then, the RNA was fragmented with the interrupt buffer, random N6 primers were reverse-transcribed, and then double-stranded cDNA was synthesized. The resultant double-stranded cDNA ends were flattened and phosphorylated at the 5′ end, with the 3′ end forming the A sticky tail with the protrusion of “A”, and then the A bubbling junction was connected to “T” protruding from the 3′ end. In the cDNA synthesis step, first-strand cDNA was synthesized using random hexamer-primed reverse transcription, followed by second-strand cDNA synthesis. The synthesized cDNA was subjected to end repair and then 3′ adenylated. Adaptors were ligated to the ends of the 3′ adenylated cDNA. The PCR products were purified with SPRI beads and then denatured by heat. The single-strand DNA was cyclized by splint oligo and DNA ligase. The RNA-Seq libraries were sequenced using the BGISEQ-500 sequencer. The raw sequencing data have been uploaded to NCBI (SRA, accession number PRJNA661708).

### 4.4. RNA-Seq Data Processing Analysis

To obtain clean reads, SOAPnuke (v1.4.0) and Trimmomatic (v0.36) were used to statistically analyze and filter the raw reads: reads that had adaptors, more than 5% unknown bases (N), or more than 20% of bases with quality less than 15 were removed. The filtered “Clean Reads” were saved in the FASTQ format and were assembled using Trinity (v2.0.6), where gene family clustering with Tgicl was performed to obtain the final unigenes. The candidate coding regions in unigenes were identified by TransDecoder (v3.0.1, https://transdecoder.github.io). MISA (v1.0, http://pgrc.ipk-gatersleben.de/misa) was used to detect unigenes. For gene functional annotation, Blastn (v2.2.23) and Diamond (v0.8.31) were used to align unigenes to NT, NR, KOG, KEGG, and SwissProt databases. For annotation, Blast2GO (v2.5.0) with NR annotation was used for GO annotation, and InterProScan5 (v5.11-51.0) was used for InterPro annotation. To identify transcription factors (TFs), getorf (version: EMBOSS:6.5.7.0) was used to find the ORFs of each unigene, and then the ORFs were aligned to TF domains (from PlnTFDB, http://plntfdb.bio.uni-potsdam.de) using hmmsearch (v3.0, http://hmmer.org). The high-quality clean reads were mapped to reference gene sequences using Bowtie2 (version 2.2.5), and then the gene expression level was calculated with RSEM (version 1.2.12). Three biological replicates of all three materials were pooled using DEGseq (parameters: fold change ≥ 2.00 and adjusted *p* value ≤ 0.05). DEGs were evaluated by hierarchical clustering using the pheatmap R package (version 1.0.12). The princomp function in R software was used for PCA (principal component analysis), and the ggplot2 package in R software was used for drawing graphs.

### 4.5. Quantitative Real-Time PCR for RNA-Seq Validation

Total RNA was reverse-transcribed into cDNA using a TransScript^®^ II One-Step gDNA Removal Kit and cDNA Synthesis SuperMix (Trans, Beijing, China). RT-qPCR experiments were performed to validate DEG expression levels by the 2^−∆∆*C*t^ method, for which TUB, CYP, and PEPKR1 were used as endogenous reference genes [30]. Using a system reaction of 15 μL was used, which contained 7.5 μL SYBR Green PCR master mix (Takara, China). Two microliters of cDNA was added to each system, 0.5 μL of each primer, and the rest were supplemented with RNase/DNase-free H2O. RT-PCR was performed using the StepOnePlus Real-time PCR System (Thermofisher, US) with a 96 well plate. PCR procedure was set as follows: 95 °C for 30 s, followed by 40 cycles of 95 °C for 15 s for denaturation, and then 60 °C for 30 s for annealing. All of the specific primers were designed with Primer 5 (Table S1).

### 4.6. Enzyme Activity and MDA Content Assays

Kenaf anthers at different stages (mononuclear, dinuclear, and mature pollen grain) were harvested from P9B, natural mutant P9BS, and P9SA for measurements. Took 0.5 g anthers and put them into the mortar. The anthers were ground in a precooled phosphate buffer (0.05 M, pH 7.8). After centrifugation for 2 times for 10 min, the supernatant was taken from the obtained abrasive tissue fluid (12,000× *g*) and placed on ice for further determination of MDA content, SOD, and POD activities. The MDA contents were detected using previously published methods [75]. Peroxidase (POD) and superoxide (SOD) activities were measured according to a previously published standard [76].

## 5. Conclusions

In this study, anther development in P9B and male-sterile lines (P9BS and P9SA) differed at the mononuclear stage. Additionally, in P9BS and P9SA, the electron transport chain was blocked due to inhibited AsA and Grx metabolism, and the activities of related enzymes were lower than those in P9B, which caused ROS to accumulate and led to abnormal PCD of tapetal cells. The tapetum supplied insufficient energy for microspore development and prevented the release of sporopollenin for pollen wall formation. Due to the lack of nutrients and energy, and incomplete pollen, the microspores were shrunken. This further affected the expression of PME/PMEI, among other related genes, leading to pollen abortion. Many previously reported TFs involved in abnormal degradation of the tapetum and cellular antioxidants, such as MYB, MAD-box, and LIM, were also affected. An ROS burst may be the direct reason for pollen abortion, and there may be a critical level of ROS accumulation that leads to the rearrangement of mitochondrial genes in P9SA, imparting it with the characteristics of CMS. The peroxidase activity and response to oxidative stress were significantly different between P9B and P9SA. This novel report regarding the natural transformation of GMS and CMS could provide new insights for further research in this area.

## Figures and Tables

**Table 1:** Overview of the de novo assembly.

Categories	P9SA	P9BS	P9B
Total raw reads (Gb)	20.40	20.57	20.41
Clean Reads Ratio (%)	96.24		
Total clean reads (Gb)	19.59	19.86	19.61
Total transcripts	588,381		
Average length (transcripts)	1281		
N50 (transcripts)	1862		
Total unigenes	125,778		
Total length (unigenes)	217,394,220		
Average length (unigenes)	1728		
N50 (unigenes)	2325		
GC (%)	42.13		

**Table 2:** Source of materials and procedure for backcross generation development.

Generation	Fertile	Sterility	Totally	Actual Separation Ratio	Expected Separation Ratio
P1 (P9B)	120	0			
P2 (P9BS)	0	5			
F1 (P2 × P1)	68	70	138	1:1.03	1:1
F2 (F1_S_ × F1_F_)	48	50	98	1:1.04	1:1
BC_1_ (F2_S_ × P1)	50	60	110	1:1.2	
BC_2_ (BC_1S_ × P1)	48	60	108	1:1.25	
BC_7_ (BC_6_ × P1)	0	96	96		
BC_8_ (BC_7_ × P1)	0	132	132		

## Data Availability

Not applicable.

## Supplementary Materials

The following are available online at https://www.mdpi.com/1422-0067/22/3/1107/s1, Figure S1:Identification of kenaf cytoplasm type, Table S1: Primer sequences used for qRT-PCR verification, Table S2: GO enrichment analysis of DEGs in the P9BS vs. P9SA, Table S3: GO enrichment analysis of DEGs in the P9B vs. P9SA, Table S4: GO enrichment analysis of DEGs in the P9B vs. P9BS, Table S5: DEGs related to respiratory chain, TCA, and ROS enzymatic scavenging between the three comparisons.

## Abbreviations

CMS	Cytoplasmic male sterility
ROS	Reactive oxygen species
DEGs	Differentially expressed genes
POD	Peroxidase
SOD	Superoxide dismutase
MDA	Malondialdehyde
MS	Male sterility
