# Supplementary material for: Reactive Oxygen Species Accumulation Strongly Allied with Genetic Male Sterility Convertible to Cytoplasmic Male Sterility in Kenaf
Source: Int J Mol Sci. 2021 Jan 23;22(3):1107. doi: 10.3390/ijms22031107 (PMC7866071; doi:10.3390/ijms22031107)
Supplement: Supplementary file 1 [file ijms-22-01107-s001.zip › Supplementary/Supplementary table 1.docx]

**Supplementary table 1**. Primer sequences used for qRT-PCR verification

|  | Gene ID | F | R |
| --- | --- | --- | --- |
| up-regulated | CL10996.Contig4_All | ATCTCCCTTCTGGGTTGTGTCCT | TGCTTTCCTCTTTTCAATCTATG |
|  | CL11265.Contig3_All | TCCTTCCCGAACCCCACC | CTCCCCGAAAGTCCCTAC |
|  | CL1289.Contig7_All | AAAACCTTCTCTTTCTCCCTCTTC | TTCTCCATTGGTGTAACCCTCTGA |
|  | Unigene21025_All | ACCTTGTCTCTTCGTATGTG | GATTGATTTTCAGGGTGTGG |
|  | CL10902.Contig2_All | CAAAAACAAAACGAGATGAA | ATGAAAGATGGAAGAGAATG |
|  | Unigene15206_All | TTACAGTATGCGAACAACC | ACCAAATAAGTCTAAAGGC |
| down-regulated | Unigene8468_All | TCTTTCAAGTCAGCACGG | AGCATAACCAAAATCTAC |
|  | CL6364.Contig9_All | ACTGACACGACACCATCTT | ATTCCATCTCCCGAACCTT |
|  | CL7119.Contig1_All | GCTGTTTTGGCTACTGGG | TCTTCGGCATCATCTTCT |
|  | CL12068.Contig3_All | GGCGGGAGTTGTGATAGTG | TCATCTGCGTAGGGTCTTT |
|  | Unigene25847_All | ATACATCCTTATTTTGGC | GTTTCACCGTCTTCACTT |
|  | CL1935.Contig3_All | AACGGCTCCCCATCTACT | CTCATTGCTGGTTCTCAC |
