# Supplementary material for: Reactive Oxygen Species Accumulation Strongly Allied with Genetic Male Sterility Convertible to Cytoplasmic Male Sterility in Kenaf
Source: Int J Mol Sci. 2021 Jan 23;22(3):1107. doi: 10.3390/ijms22031107 (PMC7866071; doi:10.3390/ijms22031107)
Supplement: Supplementary file 1 [file ijms-22-01107-s001.zip › Supplementary/Supplementary table 2.docx]

**Supplementary table 2** GO enrichment analysis of DEGs in the P9BS vs. P9SA

| GO Term ID | GO Term | Number | Rich Ratio | P value |
| --- | --- | --- | --- | --- |
| GO:0005509 | calcium ion binding | 310 | 0.227273 | 1.44E-07 |
| GO:0016491 | oxidoreductase activity | 262 | 0.215993 | 6.05E-05 |
| GO:0004857 | enzyme inhibitor activity | 105 | 0.549738 | 5.32E-32 |
| GO:0003779 | actin binding | 98 | 0.275281 | 1.03E-06 |
| GO:0030599 | pectinesterase activity | 88 | 0.567742 | 1.88E-28 |
| GO:0045330 | aspartyl esterase activity | 88 | 0.567742 | 1.88E-28 |
| GO:0005516 | calmodulin binding | 81 | 0.237537 | 0.00149 |
| GO:0005096 | GTPase activator activity | 79 | 0.262458 | 6.47E-05 |
| GO:0022857 | transmembrane transporter activity | 64 | 0.270042 | 0.000127 |
| GO:0015095 | magnesium ion | 48 | 0.338028 | 1.53E-06 |
| GO:0016760 | cellulose synthase (UDP-forming) activity | 47 | 0.26257 | 0.001731 |
| GO:0004650 | polygalacturonase activity | 43 | 0.34127 | 3.94E-06 |
| GO:0051015 | actin filament binding | 32 | 0.347826 | 4.19E-05 |
| GO:0045735 | nutrient reservoir activity | 28 | 0.405797 | 4.52E-06 |
| GO:0004373 | glycogen (starch) synthase activity | 27 | 0.375 | 3.62E-05 |
| GO:0003978 | UDP-glucose 4-epimerase activity | 26 | 0.317073 | 0.00106 |
| GO:0030570 | pectate lyase activity | 23 | 0.46 | 2.48E-06 |
| GO:0004491 | methylmalonate-semialdehyde dehydrogenase (acylating) activity | 19 | 0.527778 | 1.37E-06 |
| GO:0080019 | fatty-acyl-CoA reductase (alcohol-forming) activity | 18 | 0.4 | 0.000276 |
| GO:0102965 | alcohol-forming fatty acyl-CoA reductase activity | 18 | 0.4 | 0.000276 |
|  |  |  |  |  |
|  |  |  |  |  |
| GO Term ID | GO Term | Number | Rich Ratio | P value |
| GO:0003983 | UTP:glucose-1-phosphate uridylyltransferase activity | 16 | 0.516129 | 1.37E-05 |
| GO:0008184 | glycogen phosphorylase activity | 11 | 0.478261 | 0.000722 |
| GO:0102250 | linear malto-oligosaccharide phosphorylase activity | 11 | 0.478261 | 0.000722 |
| GO:0102499 | SHG alpha-glucan phosphorylase activity | 11 | 0.478261 | 0.000722 |
| GO:0004349 | glutamate 5-kinase activity | 10 | 0.555556 | 0.00027 |
| GO:0004350 | glutamate-5-semialdehyde dehydrogenase activity | 10 | 0.555556 | 0.00027 |
| GO:0004310 | farnesyl-diphosphate farnesyltransferase activity | 9 | 0.5 | 0.001495 |
| GO:0051996 | squalene synthase activity | 9 | 0.5 | 0.001495 |
| GO:0030598 | rRNA N-glycosylase activity | 4 | 1 | 0.000898 |
| GO:0050126 | N-carbamoylputrescine amidase activity | 4 | 1 | 0.000898 |
| GO:0090729 | toxin activity | 4 | 1 | 0.000898 |
| GO:0016021 | integral component of membrane | 4044 | 0.185284 | 1.08E-16 |
| GO:0005576 | extracellular region | 171 | 0.307002 | 1.55E-15 |
| GO:0005618 | cell wall | 139 | 0.299569 | 4.89E-12 |
| GO:0009507 | chloroplast | 117 | 0.25 | 8.81E-06 |
| GO:0005769 | early endosome | 48 | 0.338028 | 1.05E-06 |
| GO:0005856 | cytoskeleton | 47 | 0.460784 | 1.05E-11 |
| GO:0015629 | actin cytoskeleton | 25 | 0.423729 | 4.59E-06 |
| GO:0009501 | amyloplast | 22 | 0.37931 | 0.000126 |
|  |  |  |  |  |
| GO Term ID | GO Term | Number | Rich Ratio | P value |
| GO:0009706 | chloroplast inner membrane | 10 | 0.526316 | 0.000435 |
| GO:0005975 | carbohydrate metabolic process | 274 | 0.234188 | 9.77E-10 |
| GO:0071555 | cell wall organization | 134 | 0.222222 | 0.000222 |
| GO:0006952 | defense response | 120 | 0.236686 | 2.89E-05 |
| GO:0045490 | pectin catabolic process | 110 | 0.539216 | 4.52E-34 |
| GO:0006629 | lipid metabolic process | 105 | 0.224839 | 0.00065 |
| GO:0042545 | cell wall modification | 88 | 0.567742 | 9.09E-30 |
| GO:0030244 | cellulose biosynthetic process | 67 | 0.265873 | 4.47E-05 |
| GO:0006869 | lipid transport | 39 | 0.354545 | 1.44E-06 |
| GO:0019252 | starch biosynthetic process | 37 | 0.381443 | 3.34E-07 |
| GO:0009607 | response to biotic stimulus | 33 | 0.3 | 0.000368 |
| GO:0007205 | protein kinase C-activating G-protein coupled receptor signaling pathway | 29 | 0.295918 | 0.00103 |
| GO:0030042 | actin filament depolymerization | 25 | 0.423729 | 2.84E-06 |
| GO:0051017 | actin filament bundle assembly | 21 | 0.403846 | 4.17E-05 |
| GO:0005978 | glycogen biosynthetic process | 21 | 0.362069 | 0.000263 |
| GO:0006011 | UDP-glucose metabolic process | 16 | 0.516129 | 8.26E-06 |
| GO:0001678 | cellular glucose homeostasis | 16 | 0.363636 | 0.001303 |
| GO:0006696 | ergosterol biosynthetic process | 9 | 0.5 | 0.001128 |
| GO:0002229 | defense response to oomycetes | 6 | 0.75 | 0.000441 |
| GO Term ID | GO Term | Number | Rich Ratio | P value |
| GO:0080142 | regulation of salicylic acid biosynthetic process | 6 | 0.75 | 0.000441 |
| GO:1900426 | positive regulation of defense response to bacterium | 6 | 0.75 | 0.000441 |
