# Supplementary material for: Reactive Oxygen Species Accumulation Strongly Allied with Genetic Male Sterility Convertible to Cytoplasmic Male Sterility in Kenaf
Source: Int J Mol Sci. 2021 Jan 23;22(3):1107. doi: 10.3390/ijms22031107 (PMC7866071; doi:10.3390/ijms22031107)
Supplement: Supplementary file 1 [file ijms-22-01107-s001.zip › Supplementary/Supplementary table 3.docx]

**Supplementary table 3** GO enrichment analysis of DEGs in the P9B vs. P9SA.

| GO Term ID | GO Term | Number | Rich Ratio | P value |
| --- | --- | --- | --- | --- |
| GO:0016021 | integral component of membrane | 11988 | 0.549253 | 1.84E-12 |
| GO:0005524 | ATP binding | 6518 | 0.5618 | 2.84E-07 |
| GO:0006351 | transcription, DNA-templated | 2220 | 0.577223 | 6.60E-10 |
| GO:0004672 | protein kinase activity | 2182 | 0.570458 | 8.01E-05 |
| GO:0004674 | protein serine/threonine kinase activity | 1424 | 0.580514 | 3.10E-05 |
| GO:0003700 | DNA binding transcription factor activity | 1372 | 0.581602 | 2.76E-05 |
| GO:0005509 | calcium ion binding | 834 | 0.611437 | 6.28E-08 |
| GO:0016491 | oxidoreductase activity | 685 | 0.564716 | 0.049244 |
| GO:0043565 | sequence-specific DNA binding | 663 | 0.6 | 3.71E-05 |
| GO:0005576 | extracellular region | 345 | 0.61939 | 2.19E-05 |
| GO:0006952 | defense response | 316 | 0.623274 | 1.58E-05 |
| GO:0005618 | cell wall | 293 | 0.631466 | 1.07E-05 |
| GO:0005874 | microtubule | 284 | 0.606838 | 0.000738 |
| GO:0006950 | response to stress | 254 | 0.633416 | 2.06E-05 |
| GO:0003779 | actin binding | 242 | 0.679775 | 6.04E-08 |
| GO:0045490 | pectin catabolic process | 164 | 0.803922 | 3.50E-16 |
| GO:0004601 | peroxidase activity | 154 | 0.7 | 9.91E-07 |
| GO:0004857 | enzyme inhibitor activity | 151 | 0.790576 | 5.30E-13 |
| GO:0042545 | cell wall modification | 130 | 0.83871 | 6.20E-16 |
| GO:0030599 | pectinesterase activity | 130 | 0.83871 | 4.09E-15 |
| GO:0045330 | aspartyl esterase activity | 130 | 0.83871 | 4.09E-15 |
| GO:0006979 | response to oxidative stress | 126 | 0.65285 | 0.000403 |
| GO:0016788 | hydrolase activity, acting on ester bonds | 123 | 0.679558 | 9.87E-05 |
| GO Term ID | GO Term | Number | Rich Ratio | P value |
| GO:0005856 | cytoskeleton | 78 | 0.764706 | 1.15E-06 |
| GO:0006730 | one-carbon metabolic process | 77 | 0.785714 | 1.52E-07 |
| GO:0042744 | hydrogen peroxide catabolic process | 77 | 0.754902 | 2.70E-06 |
| GO:0016459 | myosin complex | 76 | 0.71028 | 0.000136 |
| GO:0003774 | motor activity | 76 | 0.71028 | 0.000249 |
| GO:0007186 | G-protein coupled receptor signaling pathway | 58 | 0.734177 | 0.000173 |
| GO:0030276 | clathrin binding | 58 | 0.74359 | 0.000183 |
| GO:0030136 | clathrin-coated vesicle | 52 | 0.722222 | 0.000798 |
| GO:0015369 | calcium:proton antiporter activity | 44 | 0.758621 | 0.000519 |
| GO:0030042 | actin filament depolymerization | 44 | 0.745763 | 0.000589 |
| GO:0015629 | actin cytoskeleton | 44 | 0.745763 | 0.000651 |
| GO:0031683 | G-protein beta/gamma-subunit complex binding | 36 | 0.8 | 0.000272 |
| GO:0006556 | S-adenosylmethionine biosynthetic process | 34 | 0.894737 | 1.85E-06 |
| GO:0004478 | methionine adenosyltransferase activity | 34 | 0.894737 | 3.16E-06 |
| GO:0030658 | transport vesicle membrane | 33 | 0.785714 | 0.000636 |
| GO:2000028 | regulation of photoperiodism, flowering | 31 | 0.794872 | 0.000596 |
| GO:0031225 | anchored component of membrane | 26 | 0.866667 | 0.000122 |
| GO:0010215 | cellulose microfibril organization | 25 | 0.862069 | 0.000186 |
| GO:0032040 | small-subunit processome | 18 | 0.9 | 0.000565 |
