# Supplementary material for: Reactive Oxygen Species Accumulation Strongly Allied with Genetic Male Sterility Convertible to Cytoplasmic Male Sterility in Kenaf
Source: Int J Mol Sci. 2021 Jan 23;22(3):1107. doi: 10.3390/ijms22031107 (PMC7866071; doi:10.3390/ijms22031107)
Supplement: Supplementary file 1 [file ijms-22-01107-s001.zip › Supplementary/Supplementary table 4.docx]

**Supplementary table 4** GO enrichment analysis of DEGs in the P9B vs. P9BS

| GO Term ID | GO Term | Number | Rich Ratio | P value |
| --- | --- | --- | --- | --- |
| GO:0003677 | DNA binding | 3053 | 0.58802 | 2.59E-08 |
| GO:0006351 | transcription, DNA-templated | 2273 | 0.591004 | 3.07E-09 |
| GO:0003700 | DNA binding transcription factor activity | 1395 | 0.591352 | 4.72E-05 |
| GO:0016491 | oxidoreductase activity | 702 | 0.57873 | 0.031388 |
| GO:0043565 | sequence-specific DNA binding | 688 | 0.622624 | 9.70E-07 |
| GO:0005576 | extracellular region | 349 | 0.626571 | 0.000148 |
| GO:0008017 | microtubule binding | 331 | 0.628083 | 0.000229 |
| GO:0006952 | defense response | 319 | 0.629191 | 9.95E-05 |
| GO:0005874 | microtubule | 300 | 0.641026 | 3.91E-05 |
| GO:0005618 | cell wall | 297 | 0.640086 | 5.00E-05 |
| GO:0008289 | lipid binding | 273 | 0.639344 | 0.000147 |
| GO:0006950 | response to stress | 254 | 0.633416 | 0.000264 |
| GO:0003779 | actin binding | 241 | 0.676966 | 9.48E-07 |
| GO:0045490 | pectin catabolic process | 168 | 0.823529 | 6.43E-17 |
| GO:0004857 | enzyme inhibitor activity | 154 | 0.806283 | 1.30E-13 |
| GO:0042545 | cell wall modification | 129 | 0.832258 | 5.18E-14 |
| GO:0030599 | pectinesterase activity | 129 | 0.832258 | 1.32E-13 |
| GO:0045330 | aspartyl esterase activity | 129 | 0.832258 | 1.32E-13 |
| GO:0016788 | hydrolase activity, acting on ester bonds | 122 | 0.674033 | 0.000537 |
| GO:0006334 | nucleosome assembly | 103 | 0.682119 | 0.000481 |
| GO:0006869 | lipid transport | 83 | 0.754545 | 5.41E-06 |
| GO:0005856 | cytoskeleton | 75 | 0.735294 | 9.07E-05 |
| GO:0006730 | one-carbon metabolic process | 73 | 0.744898 | 4.19E-05 |
| GO:0045735 | nutrient reservoir activity | 52 | 0.753623 | 0.00043 |
|  |  |  |  |  |
| GO Term ID | GO Term | Number | Rich Ratio | P value |
| GO:0030042 | actin filament depolymerization | 45 | 0.762712 | 0.000507 |
| GO:0015629 | actin cytoskeleton | 45 | 0.762712 | 0.000604 |
| GO:0030570 | pectate lyase activity | 40 | 0.8 | 0.000223 |
| GO:0006556 | S-adenosylmethionine biosynthetic process | 33 | 0.868421 | 2.60E-05 |
| GO:0004478 | methionine adenosyltransferase activity | 33 | 0.868421 | 3.33E-05 |
| GO:0031225 | anchored component of membrane | 26 | 0.866667 | 0.000244 |
| GO:0043044 | ATP-dependent chromatin remodeling | 26 | 0.83871 | 0.000633 |
| GO:0010215 | cellulose microfibril organization | 25 | 0.862069 | 0.000342 |
| GO:0016998 | cell wall macromolecule catabolic process | 24 | 0.857143 | 0.000543 |
| GO:0003678 | DNA helicase activity | 13 | 1 | 0.000442 |
