# Supplementary material for: Reactive Oxygen Species Accumulation Strongly Allied with Genetic Male Sterility Convertible to Cytoplasmic Male Sterility in Kenaf
Source: Int J Mol Sci. 2021 Jan 23;22(3):1107. doi: 10.3390/ijms22031107 (PMC7866071; doi:10.3390/ijms22031107)
Supplement: Supplementary file 1 [file ijms-22-01107-s001.zip › Supplementary/Supplementary table 5.docx]

**Supplementary table 5** DEGs related to respiratory chain, TCA, and ROS enzymatic scavenging between the three comparisons

| Gene ID | GO Term | log2(A/B) | Qvalue | Pvalue |
| --- | --- | --- | --- | --- |
| CL10146.Contig1_All | [P]GO:0015986//ATP synthesis coupled proton transport | -3.51143 | 0.004669 | 0.025276 |
| CL10146.Contig2_All | [P]GO:0015986//ATP synthesis coupled proton transport | -5.39429 | 0 | 0 |
| CL4887.Contig1_All | [P]GO:0015986//ATP synthesis coupled proton transport | -8.17181 | 0 | 0 |
| CL4887.Contig2_All | [P]GO:0015986//ATP synthesis coupled proton transport | -8.89566 | 2.79E-47 | 4.63E-47 |
| Unigene7166_All | [P]GO:0015986//ATP synthesis coupled proton transport | -7.51836 | 3.45E-22 | 8.68E-22 |
| CL8396.Contig2_All | [F] GO:0050664//oxidoreductase activity, acting on NAD(P)H, oxygen as acceptor; GO:0004601//peroxidase activity; | -10.1247 | 0 | 0 |
| CL8419.Contig2_All | [P]GO:0042744//hydrogen peroxide catabolic process; GO:0006979//response to oxidative stress; GO:0004601//peroxidase activity | 2.83529 | 2.45E-23 | 6.01E-23 |
| Unigene15808_All | [P]GO:0042744//hydrogen peroxide catabolic process; GO:0006979//response to oxidative stress; GO:0004601//peroxidase activity; | -10.6138 | 0 | 0 |
| Unigene2723_All | [F]GO:0005509//calcium ion binding; GO:0004601//peroxidase activity; GO:0050664//oxidoreductase activity, acting on NAD(P)H, oxygen as acceptor; | -7.20739 | 3.53E-35 | 6.96E-35 |
| Unigene6255_All | [P]GO:0042744//hydrogen peroxide catabolic process;GO:0090378//seed trichome elongation;GO:0042542//response to hydrogen peroxide; [C]GO:0046861//glyoxysomal membrane;GO:0016021//integral component of membrane;[F] binding;GO:0016688//L-ascorbate peroxidase activity; | -8.64225 | 1.81E-117 | 1.57E-117 |
| Gene ID | GO Term | log2(A/B) | Qvalue | Pvalue |
| CL5810.Contig4_All | [P]GO:0006801//superoxide metabolic process; | -7.78397 | 0 | 0 |
| CL2649.Contig3_All | [P]GO:0005975//carbohydrate metabolic process; GO:0006099//tricarboxylic acid cycle; GO:0006108//malate metabolic process; [F]GO:0030060//L-malate dehydrogenase activity; | -5.47432 | 2.41E-07 | 9.55E-07 |
| CL1920.Contig4_All | [P]GO:0006099//tricarboxylic acid cycle; GO:0008177//succinate dehydrogenase (ubiquinone) activity; | -5.85517 | 1.40E-243 | 6.70E-244 |
| Unigene24034_All | [P]GO:0006099//tricarboxylic acid cycle;GO:0015977//carbon fixation;GO:0048366//leaf development;GO:0016036//cellular response to phosphate starvation;GO:0090377//seed trichome initiation;GO:0090378//seed trichome elongation;GO:0051262//protein tetramerization;[C]GO:0016020//membrane;GO:0005829//cytosol;GO:0048046//apoplast;[F]GO:0008964//phosphoenolpyruvate carboxylase activity; | -6.67902 | 0 | 0 |
| Unigene14979_All | [P]GO:0006099//tricarboxylic acid cycle;[F]GO:0030976//thiamine pyrophosphate binding;GO:0004591//oxoglutarate dehydrogenase (succinyl-transferring) activity; | -7.19083 | 0 | 0 |
| Unigene15471_All | GO:0006099//tricarboxylic acid cycle; GO:0005975//carbohydrate metabolic process; [F]GO:0030060//L-malate dehydrogenase activity; | -7.21291 | 0 | 0 |
| Unigene14981_All | [P]GO:0006099//tricarboxylic acid cycle; [F]GO:0030976//thiamine pyrophosphate binding; GO:0004591//oxoglutarate dehydrogenase; | -8.4079 | 0 | 0 |
| CL11419.Contig1_All | [P]GO:0006099//tricarboxylic acid cycle;[F]GO:0004449//isocitrate dehydrogenase (NAD+) activity; | -9.63343 | 0 | 0 |
| CL6178.Contig1_All | [P]GO:0006099//tricarboxylic acid cycle; [F]GO:0046912//transferase activity, transferring acyl groups, acyl groups converted into alkyl on transfer; | -10.0535 | 3.71E-88 | 4.00E-88 |
| Unigene14980_All | [P]GO:0006099//tricarboxylic acid cycle; [F]GO:0004591//oxoglutarate dehydrogenase (succinyl-transferring) activity; GO:0030976//thiamine pyrophosphate binding; | -10.5264 | 0 | 0 |
| Unigene24029_All | [P]GO:0051262//protein tetramerization;GO:0090377//seed trichome initiation;GO:0006099//tricarboxylic acid cycle;GO:0090378//seed trichome elongation;GO:0016036//cellular response to phosphate starvation;GO:0048366//leaf development;GO:0015977//carbon fixation;[C]GO:0016020//membrane;GO:0048046//apoplast;GO:0005829//cytosol;[F]GO:0008964//phosphoenolpyruvate carboxylase activity; | -10.5831 | 0 | 0 |
