# Supplementary figures and images for: Reactive Oxygen Species Accumulation Strongly Allied with Genetic Male Sterility Convertible to Cytoplasmic Male Sterility in Kenaf
Source: Int J Mol Sci. 2021 Jan 23;22(3):1107. doi: 10.3390/ijms22031107 (PMC7866071; doi:10.3390/ijms22031107)

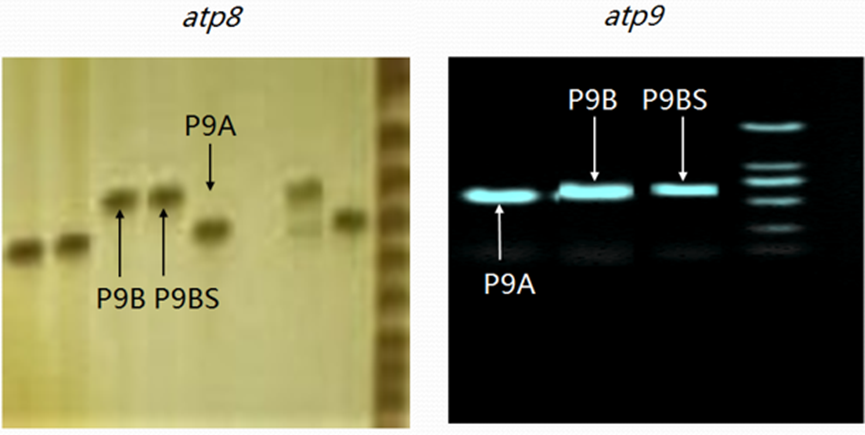

Supplement: Supplementary file 1 [file ijms-22-01107-s001.zip › Supplementary/Supplementary Figure 1.tif]
